# Host hybridization as a potential mechanism of lateral symbiont transfer in deep‐sea vesicomyid clams

**DOI:** 10.1111/mec.15224

**Published:** 2019-09-23

**Authors:** Corinna Breusing, Shannon B. Johnson, Robert C. Vrijenhoek, Curtis R. Young

**Affiliations:** ^1^ Monterey Bay Aquarium Research Institute Moss Landing CA USA; ^2^ National Oceanography Centre Southampton UK

**Keywords:** horizontal transfer, hybridization, symbiosis, vertical transmission, vesicomyid clams

## Abstract

Deep‐sea vesicomyid clams live in mutualistic symbiosis with chemosynthetic bacteria that are inherited through the maternal germ line. On evolutionary timescales, strictly vertical transmission should lead to cospeciation of host mitochondrial and symbiont lineages; nonetheless, examples of incongruent phylogenies have been reported, suggesting that symbionts are occasionally horizontally transmitted between host species. The current paradigm for vesicomyid clams holds that direct transfers cause host shifts or mixtures of symbionts. An alternative hypothesis suggests that hybridization between host species might explain symbiont transfers. Two clam species, *Archivesica gigas* and *Phreagena soyoae*, frequently co‐occur at deep‐sea hydrocarbon seeps in the eastern Pacific Ocean. Although the two species typically host gammaproteobacterial symbiont lineages marked by divergent *16S* rRNA phylotypes, we identified a number of clams with the *A. gigas* mitotype that hosted symbionts with the *P. soyoae* phylotype. Demographic inference models based on genome‐wide SNP data and three Sanger sequenced gene markers provided evidence that *A. gigas* and *P. soyoae* hybridized in the past, supporting the hypothesis that hybridization might be a viable mechanism of interspecific symbiont transfer. These findings provide new perspectives on the evolution of vertically transmitted symbionts and their hosts in deep‐sea chemosynthetic environments.

## Abstract 2

https://doi.org/10.1111/mec.15262

## 1 INTRODUCTION

Host–microbe symbioses are universal phenomena that are now considered key drivers of evolutionary innovation (Archibald, 2015; Brucker & Bordenstein, 2012; McFall‐Ngai, 2008; McFall‐Ngai et al., 2013). Over the past several decades, it has been established that symbiotic associations led to the evolution of cellular organelles and eukaryotic cell life (Archibald, 2015), while recent studies have emphasized their role in the formation of species (Brucker & Bordenstein, 2012). Transmission of microbes contributes to maintenance of symbiotic relationships across host generations and differences in transmission modes have important implications for the evolution of both partners (Bright & Bulgheresi, 2013; Vrijenhoek, 2010). Under vertical transmission, symbionts are inherited through the maternal and/or—less frequently—the paternal germ line (e.g., Ebert, 2013; Moran & Dunbar, 2006; Watanabe, Yukuhiro, Matsuura, Fukatsu, & Noda, 2014). In the predominant case of uniparental maternal inheritance, symbiont and mitochondrial genomes are cotransmitted and are thus genetically and evolutionarily linked. Bottleneck effects during transovarial transmission strongly reduce the effective size and genetic diversity of the symbiont population within individual hosts, thereby increasing the fixation of slightly deleterious mutations (Rispe & Moran, 2000; Vrijenhoek, 2010). Since recombination with environmental bacteria is limited and certain symbiont gene functions become obsolete or are complemented by the host or secondary symbiotic microbes, vertically transmitted symbionts typically lose genes through drift and selection, resulting in significant reductions in genome size (Bennett & Moran, 2015; Moran, McCutcheon, & Nakabachi, 2008; Sloan & Moran, 2012). Apart from vertical transmission, symbionts can be transmitted horizontally, either through uptake of free‐living strains in the environment or through direct transfer between hosts (Bright & Bulgheresi, 2013; Ebert, 2013; Vrijenhoek, 2010). Because symbionts are acquired from a potentially diverse mixture of bacterial strains each generation, horizontal transmission often results in genetic heterogeneity in the symbiont population and the absence of co‐evolution between host and symbiont. In contrast to vertically transmitted symbionts, horizontally transmitted symbionts switch between intra‐ and extrahost life phases, which increases rates of recombination and selective pressures for retaining genes necessary for surviving outside the host environment (Vrijenhoek, 2010). In various cases, it has been shown that horizontal transmission can supplement the vertical transmission mode (Ebert, 2013), thereby providing opportunities for recombination that can counteract the ongoing genome degradation in vertically transmitted symbionts (Vrijenhoek, 2010). Despite growing research on diverse host–microbe relationships, the mechanisms of symbiont transmission and their evolutionary consequences are still poorly understood (Bright & Bulgheresi, 2013).

Deep‐sea invertebrates that inhabit hydrothermal vents, hydrocarbon seeps and sites of organic enrichment have evolved intriguing symbioses that compensate for the absence of sunlight and the trophic benefits of photosynthesis. Associations with chemoautotrophic bacteria that derive energy from the oxidation of sulphides, hydrogen or methane can support lush invertebrate communities in these unusual habitats (Dubilier, Bergin, & Lott, 2008). Clams of the family Vesicomyidae belong to the key fauna in chemosynthesis‐based ecosystems worldwide (Johnson, Krylova, Audzijonyte, Sahling, & Vrijenhoek, 2016). Lacking a functional digestive system, they rely nutritionally on their thiotrophic gammaproteobacterial symbionts that inhabit specialized cells in the gill tissue of their host. Previous histological and molecular studies showed that vesicomyid symbionts are maternally inherited and can be grouped into two different phylogenetic clades that differ in their status of genome reduction (Kuwahara et al., 2011). Clade I symbionts have highly reduced genomes that lack crucial genes for DNA recombination and repair, whereas clade II symbionts retain functional copies of these genes and have slightly larger genome sizes (Kuwahara et al., 2007, 2011; Shimamura et al., 2017).

Although maternal inheritance appears to be the main transmission route of these symbionts, rare occasions of horizontal transfer have been suggested given that host mitochondrial and symbiont *16S* rRNA phylogenies are sometimes incongruent (Ozawa et al., 2017; Stewart, Young, & Cavanaugh, 2008, 2009; Vrijenhoek, 2010). Different mechanisms have been proposed to explain how lateral acquisition of symbionts could occur in vesicomyid clams (Stewart, Young, & Cavanaugh, 2008): (a) hybridization between host species including the presence of doubly uniparental inheritance, (b) acquisition from a stable free‐living symbiont population, (c) direct transfer between hosts without the involvement of hybridization, for example through contact between eggs, contact between eggs and host tissue or uptake of symbionts that have been released from moribund clams.

Two recent studies hypothesized that direct transfer is the main mechanism leading to symbiont mixtures or displacements of native symbionts in vesicomyid clams. Decker, Olu, Arnaud‐Haond, and Duperron (2013) reported that individual vesicomyid clams from the Gulf of Guinea can host mixtures of native and non‐native symbiont phylotypes when distinct host species co‐occur in the same seep habitat. These authors argued that physical proximity could promote symbiont exchanges among very distantly related clam taxa. Ikuta et al. (2016) recently showed that the symbiont of *Phreagena okutanii* (previously *Calyptogena okutanii*) spends part of its life attached to the surface of the host's eggs, thereby strengthening Stewart et al.'s (2008) argument that direct contact between eggs or eggs and host tissues can lead to lateral symbiont transfer between co‐occurring clam species. While these studies considered the possibility of host hybridization or environmental symbiont acquisition unlikely, these hypotheses have not been directly addressed by previous analyses. Here, we present a new case of discrepant symbiont compositions in two eastern Pacific clams, *Archivesica gigas* and *Phreagena soyoae*, species that are easily distinguished based on mitochondrial and nuclear markers (Johnson et al., 2016). Using demographic inference models based on genome‐wide SNPs as well as traditional DNA markers, we investigated the hypothesis that hybridization between the two species might be a mechanism of horizontal symbiont transfer in this system and examined the nature of this gene flow between the taxa.

## 2 MATERIAL AND METHODS

### 2.1 Sample collection and preparation

**Figure 1:**
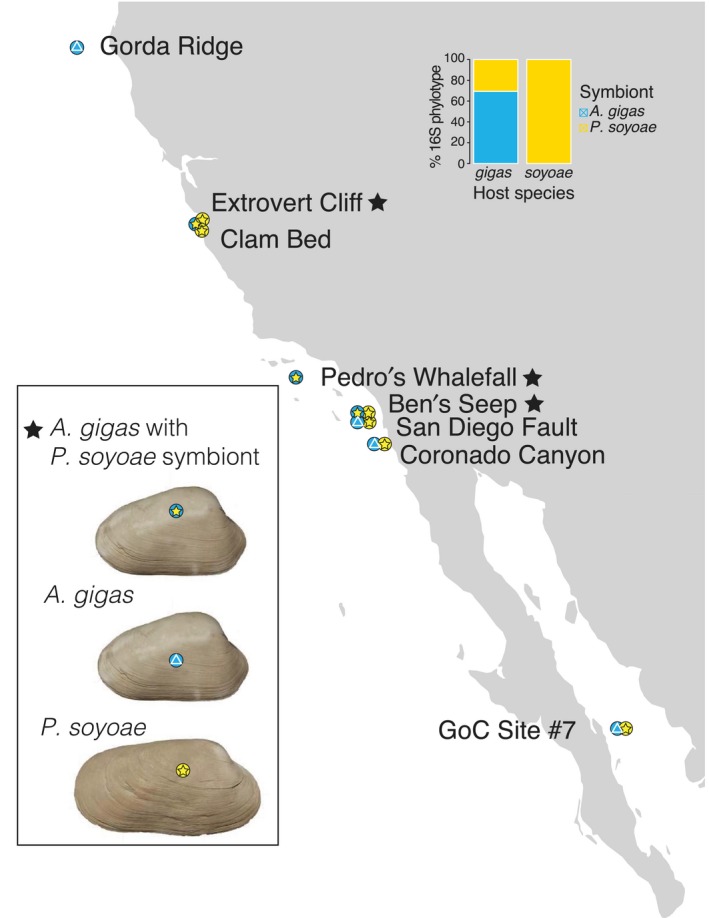
Sampling sites for clam specimens in the eastern Pacific Ocean. Blue circle = *A. gigas* host, yellow circle = *P. soyoae* host, blue triangle = *A. gigas* symbiont, yellow star = *P. soyoae* symbiont. The barplot in the upper right shows the proportion of each symbiont type in the two host species across all individuals sampled. Clam images were taken by Shannon Johnson

Clams were collected with remotely operated vehicles (ROVs) from eight eastern Pacific seep sites during cruises between 2000 and 2015 (Table 1; Figure 1). Upon recovery of the ROV, specimens were either dissected and frozen at –80°C or preserved in 95% ethanol. DNA was extracted from symbiont‐bearing gill and symbiont‐free foot or adductor muscle tissue with the QIAGEN DNeasy Blood & Tissue Kit according to manufacturer's instructions. An RNA digestion step was included as advised in the protocol. We constructed the map of sampling localities with ggmap in rstudio (https://cran.r-project.org/web/packages/ggmap/citation.html).

**Table 1:** Geographic coordinates, depths, dive numbers, sample sizes (*N*) and host species for the investigated clam sites

Locality	Lat	Lon	Depth (m)	Dive^a^	*N*	Year	Host^b^
GoC Site#7	26.75	–111.17	1,371	D369, D390	8	2012	PA
Coronado Canyon	32.36	–117.38	1,266	D766	2	2015	PA
Ben's Seep	32.90	–117.78	1,021	D472	24	2013	PA
San Diego Fault	32.91	–117.77	999	D625	5	2014	PA
Pedro's Whalefall	33.77	–119.52	1895	D464, D474	14	2013	A
Clam Bed	36.73	–122.03	905	D97	6	2009	P
Extrovert Cliff	36.77	–122.08	960	V1676, V1682 V2034, T233	28	2000–2001	PA
Gorda Ridge	40.36	–125.21	1588	T349	10	2001	A

a Submersibles: D = *Doc Ricketts*, T = *Tiburon*, V = *Ventana*.

b Host: P = *Phreagena soyoae*, A = *Archivesica gigas*.

### 2.2 Sanger sequencing of host and symbiont genes

The mitochondrial cytochrome c oxidase subunit I (*COI*), the nuclear histone 3 (*H3*) and the nuclear ADP/ATP translocase (*ANT*) genes were used for host species identification. PCR and sequencing protocols followed Johnson et al. (2016). Assembly of forward and reverse reads, multiple alignments and phasing of nuclear genes were done as in Breusing, Johnson, Tunnicliffe, and Vrijenhoek (2015). To identify the dominant symbiont lineage in the sampled clam species, we sequenced the full‐length *16S* rRNA using the universal eubacterial primers 27F/1492R (Lane, 1991). Gene amplifications and sequencing reactions were performed as in Vrijenhoek, Duhaime, and Jones (2007), while sequence analysis was done as described above.

### 2.3 ezRAD sequencing and estimation of allele frequencies

The Sanger sequence analyses indicated that several clams with the *A. gigas COI* mitotype contained the *P. soyoae* symbiont *16S* phylotype. To determine whether hybridization had occurred between the two host species and could therefore possibly account for the observed symbiont switch, we developed a SNP panel from ezRAD sequencing of five putatively pure *A. gigas* (Gorda Ridge) and five putatively pure *P. soyoae* (Clam Bed) individuals. These sites were chosen as references as they each contained only one clam species without any evidence for genetic admixture or symbiont discrepancies. The composition of these SNPs was evaluated in four clams from Pedro's Whalefall in which host–symbiont discrepancies were found. Library preparation and sequencing was performed at the Huntsman Cancer Institute at the University of Utah and UC Davis. The library preparation protocol was adapted from the original methods described in Toonen et al. (2013) and is provided in full detail in Appendix S1. Sequencing of the 14 clams was done with a 2 × 125–150 bp paired‐end protocol on Illumina HiSeq 2500 and 4000 instruments. Following quality checks with fastqc (https://www.bioinformatics.babraham.ac.uk/projects/fastqc/), the raw reads were compared against draft genome assemblies of the clam symbionts and host mitochondrial and ribosomal genes (C. R. Young, unpublished data) as well as the PhiX sequencing control to remove potential contaminants and obtain a purely nuclear gene data set (Appendix S2). Unmapped paired‐end reads were then trimmed and quality filtered with trimmomatic (Bolger, Lohse, & Usadel, 2014) and assembled in ddocent version 2.2.17 (Puritz, Hollenbeck, & Gold, 2014) following recommendations for assembly optimization at http://www.dDocent.com. Assembly parameters were adjusted as follows: clustering threshold = 0.9; minimum coverage of a read within an individual = 6; minimum number of individuals containing a unique sequence = 4. Basic quality metrics and information about the sequencing data are given in Appendix S3. Exhaustive exploration of various settings in the ddocent SNP filtering pipeline all resulted in spurious patterns in population‐specific allele frequency spectra (AFS) and poor convergence in downstream population genomic analyses. As recently shown by Warmuth and Ellegren (2019), traditional SNP calling from RADseq data can introduce bias in demographic inference. Based on these results, we used angsd version 0.920 (Korneliussen, Albrechtsen, & Nielsen, 2014) to estimate AFSs and other population genetic statistics in this study (Appendix S4). To remove low‐quality sites from the analyses, we used a minimum mapping quality of 30 (minMapQ = 30), a minimum base quality of 20 (minQ = 20) and a minimum depth of 20 (setMinDepth = 20). We further adjusted mapping quality for excessive mismatches (C = 50), removed sites with missing data, excluded spurious and improperly paired reads and computed per‐base alignment qualities (BAQ = 1) to resolve false variants that were caused by misalignments. Potentially paralogous regions were excluded by discarding reads that had multiple hits to the reference assembly and by considering only sites that had a maximum depth of 250 (which we chose as reasonable threshold based on the mean read depth distribution). Inferences were based on the folded AFS due to no outgroup information available before the analysis. The joint AFS between *A. gigas* and *P. soyoae* was calculated with the angsd subprogram realsfs and subsequently folded in ∂a∂i version 1.6.3 (Gutenkunst, Hernandez, Williamson, & Bustamante, 2009).

### 2.4 Phylogenetic and population genetic analyses

We used the program popart version 1.7 (http://popart.otago.ac.nz/) to create phylogenetic networks for the symbiont *16S* rRNA gene and the host mitochondrial and nuclear genes. Networks were generated based on the median‐joining algorithm with the epsilon parameter set to 0. Diversity and *F*_ST_ statistics for Sanger data were calculated in mega version 10.0.5 (Kumar, Stecher, Li, Knyaz, & Tamura, 2018) and genodive version 2.0b27 (Meirmans & van Tienderen, 2004), respectively. *F*_ST_ values were corrected after Benjamini and Yekutieli (2001). To obtain estimates of genomic divergence and structure, we computed pairwise *F*_ST_s, PCAs and admixture proportions from the RADseq data set using the ngstools version 1.0.2 package (Fumagalli, Vieira, Linderoth, & Nielsen, 2014) and the ngsadmix subprogram with 100,000 maximum iterations in angsd.

### 2.5 Demographic inference

We used the program ima2 (Hey, 2010) on the three Sanger sequenced genes *mtCOI*, *H3* and *ANT* to test whether introgression had occurred in the evolutionary history of the two clam species or whether shared polymorphisms were mostly a result of incomplete lineage sorting. ima2 was run under a two‐population model differentiated by *A. gigas* and *P. soyoae* genotypes. Isolation‐with‐migration analyses make several assumptions about the nature of the data, including no recombination within genes, no genetic linkage, absence of population structure and gene flow from unsampled species as well as selective neutrality. While most assumptions are robust to moderate levels of violation (Strasburg & Rieseberg, 2010), recombination can introduce significant bias into parameter estimates. To exclude recombining fragments from the analyses, we applied the four‐gamete test in the program imgc (Woerner, Cox, & Hammer, 2007). The infinite sites substitution model with an inheritance scalar of 1.0 was used for all nuclear genes and 0.25 was used for the mitochondrial *COI* locus under the HKY model. Analyses were run multiple times with at least 10^7^ steps, where the first 10^4^ steps were discarded as a burn‐in. We used geometric heating with parameters between 0.99 and 0.3 with 50 attempts at chain swapping per iteration between the 50–80 chains.

Complementary to the ima2 approach, the program ∂a∂i version 1.6.3 (Gutenkunst et al., 2009) was used to infer the demographic history of the two clam species from the folded joint allele frequency spectrum estimated from the RADseq data set. This approach is considerably more flexible than ima2 with respect to demographic models and the variability of rates among different genomic regions. As in the ima2, we defined two populations based on the respective genotypic signature. We tested seven different models of evolutionary divergence as implemented in Tine et al. (2014): strict isolation (SI), isolation‐with‐migration (IM), ancient migration (AM), secondary contact (SC), as well as IM, AM and SC with heterogeneous introgression across genomic loci (IM2M, AM2M, SC2M). All models were fitted using hot and cold annealing followed by L‐BFGS‐B optimization. For each model, we performed 80 independent runs with 5,000 iterations per optimization to find the global maximum. After excluding spurious runs where parameter estimates hit the model boundaries, the iteration with the lowest Akaike information criterion (AIC) was chosen as best fit. AIC weights (Burnham & Anderson, 2002; Stewart et al., 2008) were used to express relative support among the set of models that we examined.

## 3 RESULTS

### 3.1 Sanger sequenced genes: Haplotype diversity and differentiation in hosts and symbionts

**Figure 2:**
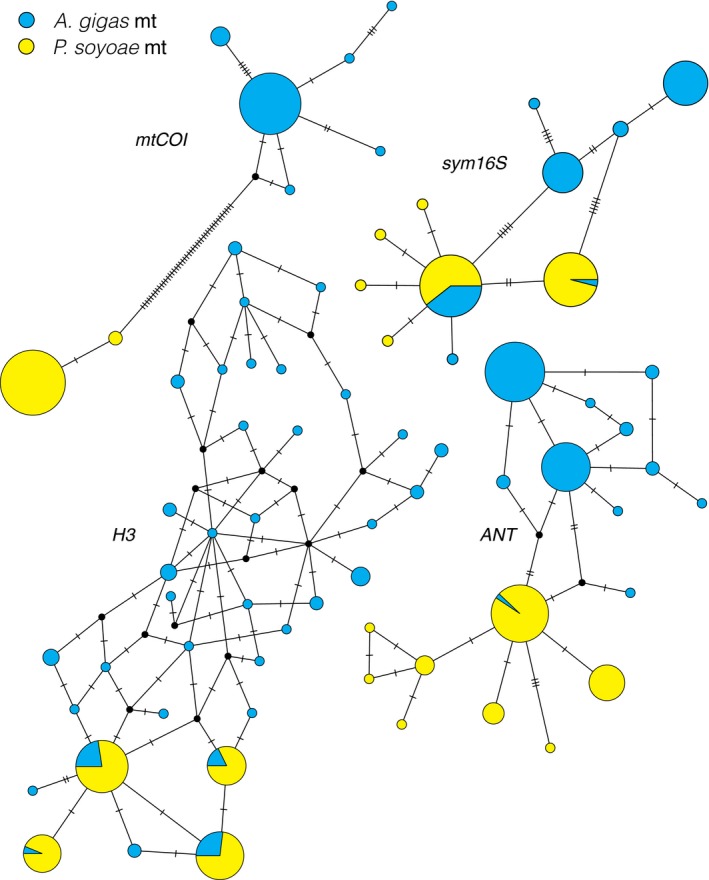
Haplotype network for host (*mtCOI*, *ANT*, *H3*) and symbiont (*sym16S*) genes. Each circle represents a single haplotype where circle size is proportional to frequency in the data set. Lines on connecting branches indicate number of mutations between haplotypes. For *ANT*, *H3* and *sym16S*, *P. soyoae*‐specific alleles (yellow) are found in *A. gigas* (blue)

Phylogenetic networks for a 518‐bp fragment of the host mitochondrial *COI* gene revealed a clear segregation of haplotypes into *A. gigas*‐ and *P. soyoae*‐specific clades (Figure 2). Haplotypes for this gene were the most divergent and were fixed between the two clam species (9.4% K2P distance). Within‐clade divergence and overall haplotype diversity (*H*) were low (*A. gigas*: 0.02% K2P; *P. soyoae*: 0.21% K2P; eight haplotypes; *H* = 0.6 ± 0.03 *SD*). Based on *mtCOI*, the *A. gigas* and *P. soyoae* populations were strongly differentiated from each other (*F*_ST_: 0.561–1.000), while no population differentiation was observed within species (Appendix S5). Although the nuclear genes *ANT* and *H3* could be grouped into *A. gigas*‐ and *P. soyoae*‐specific clades as well, some haplotypes were shared between species. In both cases, *A. gigas* contained polymorphisms that were characteristic of *P. soyoae*, whereas the opposite case was not observed. Compared to *mtCOI*, the between‐clade sequence divergence estimates for both nuclear genes were lower (0.89% K2P for *ANT* and 1.1% K2P for *H3*), while the within‐clade sequence divergence and haplotype diversity were higher (*ANT*: 0.21% K2P for *A. gigas* and 0.20% K2P for *P. soyoae*; *H* = 0.81 ± 0.0017 *SD*; *H3*: 1.2% K2P for *A. gigas* and 0.3% K2P for *P. soyoae*; *H* = 0.88 ± 0.0002 *SD*). Based on the nuclear genes, the *A. gigas* and *P. soyoae* populations were weakly to highly differentiated (*F*_ST_: 0.077–0.321), while usually no differentiation was present within species (Appendix S5). The symbiont *16S* rRNA gene mirrored the two host nuclear genes in terms of clade differentiation (0.42% K2P distance), but showed a lower diversity (*A. gigas*: 0.3% K2P; *P. soyoae*: 0.1% K2P; 11 phylotypes; *H* = 0.77 ± 0.021 *SD*).

In 15 individuals, the *P. soyoae*‐specific symbiont *16S* rRNA phylotype was found in clams that had the *A. gigas* mitotype. We observed 13 of these discrepancies at Pedro's Whalefall, where *A. gigas* was the only clam species found. The two other discrepancies were observed at Extrovert Cliff and Ben's Seep, where both species co‐occurred. In all other sequenced clams, the symbiont *16S* rRNA lineages corresponded to the host *mtCOI* lineages, as expected for symbioses with vertical transmission (Table 2).

**Table 2:** Host mitochondrial *COI* and symbiont *16S* rRNA combinations found in this study

Mitotype	Ribotype	*N*
*A. gigas*	*A. gigas*	34
*P. soyoae*	*P. soyoae*	48
*A. gigas*	*P. soyoae*	15^a^
*P. soyoae*	*A. gigas*	0

a Pedro's Whalefall: 13, Extrovert Cliff: 1, Ben's Seep: 1.

### 3.2 RADseq single nucleotide polymorphisms: Genomic divergence between clam species

**Figure 3:**
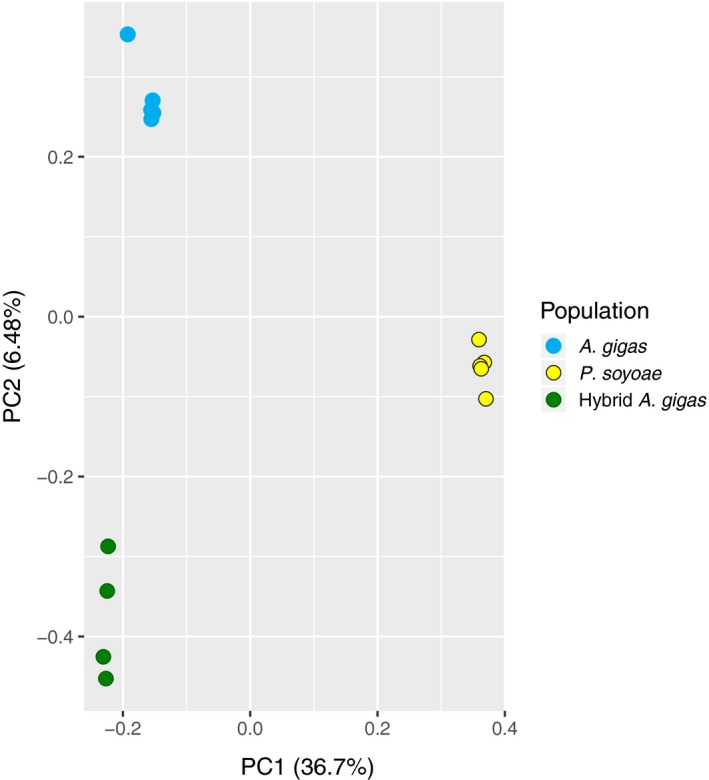
PCA plot based on the genome‐wide RADseq data set. The hybrid *A. gigas*‐like clams (green) form a separate group from the pure species (yellow, blue)

Genotype likelihood estimations in angsd resulted in a total of 349,288 shared sites for population genetic inferences. Principal component analyses based on this data set indicated a clear distinction of three different genetic groups corresponding to pure *A. gigas*, pure *P. soyoae* and the Pedro's Whalefall clams that contained the *A. gigas* mitotype but the *P. soyoae* symbiont phylotype (Figure 3). The first two principal components explained 43.18% of the variance in this data set. The PCA results were confirmed by admixture analyses that grouped all clam populations as separate entities without any evidence for recent introgression (Appendix S6). On a genome‐wide scale, the hybrid and typical *A. gigas* were weakly differentiated (*F*_ST_ = 0.105), while both of these groups showed a high divergence from *P. soyoae* (*F*_ST_ *P. soyoae*—*A. gigas*: 0.341; *F*_ST_ *P. soyoae*—hybrid *A. gigas*: 0.328).

### 3.3 Gene flow

**Figure 4:**
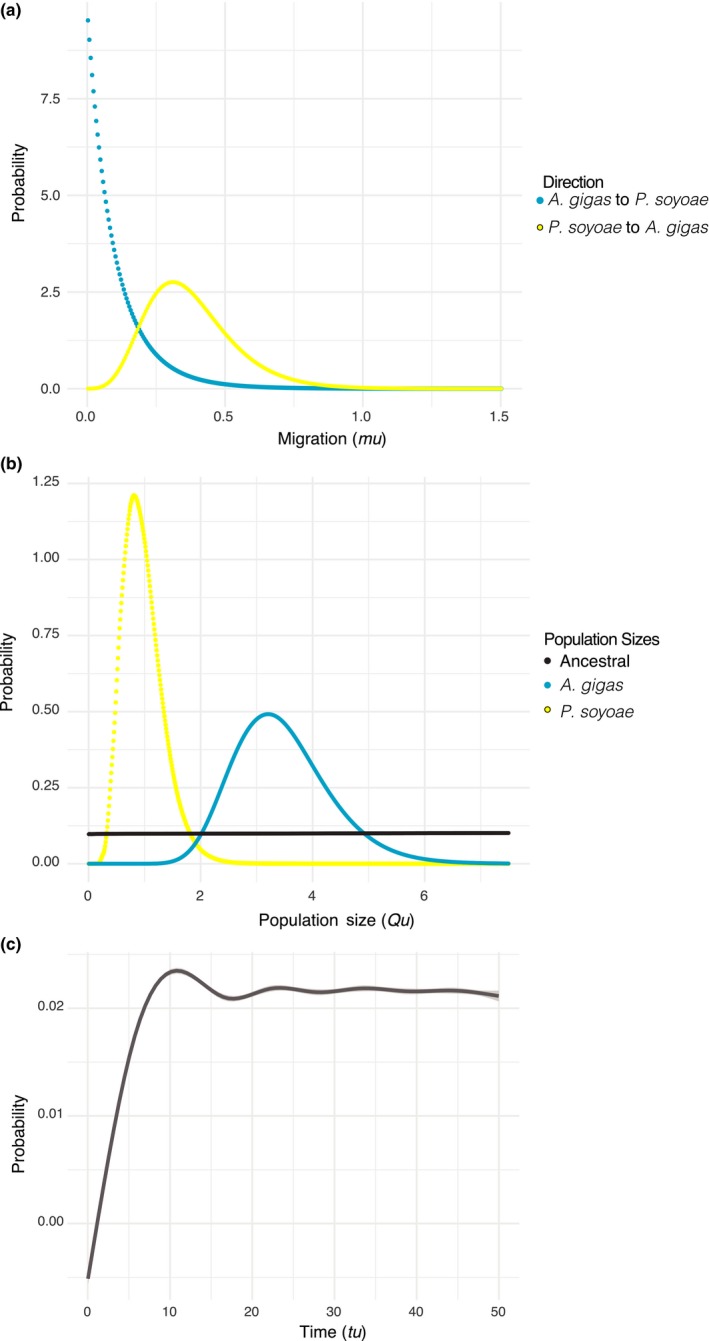
Isolation‐with‐migration analyses. (a) Migration rates indicating significant gene flow from *P. soyoae* to *A. gigas* in the evolutionary past; (b) effective population sizes; (c) time of population splitting

**Figure 5:**
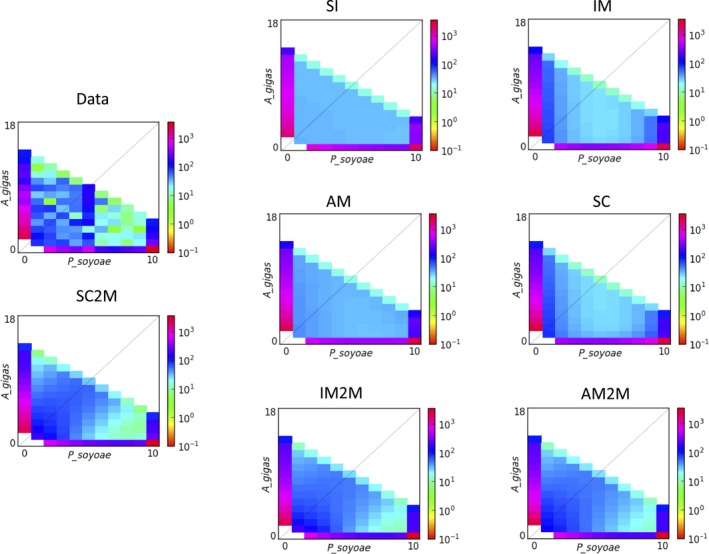
Observed and fitted joint folded allele frequency spectra as calculated in ∂a∂i. The figure shows the AFS of *A. gigas* (*x*‐axis, nine individuals) plotted against the AFS of *P. soyoae* (*y*‐axis, five individuals). The colour scheme indicates the frequencies of minor alleles in each population across all polymorphic sites. The SC2M model was the scenario with the highest likelihood, and its AFS is shown in comparison with the other tested models

Both ∂a∂i and ima2 analyses provided evidence for divergence with gene flow between the two clam species, supporting models of asymmetric migration from *P. soyoae* into *A. gigas* (Figures 4 and 5; Table 3). Despite the large phylogenetic distance between the two clam genera, the ima2 analyses could not approximate the time of population splitting or ancestral population size accurately, which indicates a lack of information to constrain these parameters due to the limited number of loci examined. ∂a∂i favoured the secondary contact model with heterogeneous gene flow (SC2M) as most likely scenario of the speciation process (Table 3; AIC weight for SC2M: 1.00; AIC weights for other models: ~0.00). This model suggested a recent secondary contact event after a comparatively long time of species divergence, resulting in (a) mainly neutral gene flow into *A. gigas* and (b) reduced migration of barrier genes between species. While the SC2M model fits the data significantly better than any other model, all models with two classes of gene flow parameters (IM2M and AM2M) were better fits to the data than those without (Table 3), and better predicted the AFS observed in *A. gigas* and *P. soyoae* (Figure 5), suggesting that accounting for differential introgression rates across the genome is useful to predict certain characteristics of our data.

**Table 3:** Best model runs from the ∂a∂i analyses. The SC2M scenario had the highest likelihood among all models tested. Model parameters are given as scaled units based on *N*_ref_, the size of the ancestral population

Model	Ln	AIC	AICw	Theta	*N*_Ag_	*N*_Ps_	*M*_Ag‐Ps_	*M*_Ps‐Ag_	Me_Ag‐Ps_	Me_Ps‐Ag_	*T*_am/sc_	*T*_s_	*P*
SI	–3586.08	7,178.16	0.00	8,621.58	0.28	0.14	–	–	–	–	–	0.19	–
IM	–2896.32	5,802.64	0.00	61,678.91	0.09	0.01	0.08	14.56	–	–	–	0.52	–
AM	–2497.97	5,007.94	0.00	58,824.98	0.08	0.01	1.65E−09	19.90	–	–	0.33	3.62E−03	–
SC	–2848.03	5,708.06	0.00	1826.07	3.23	0.23	0.01	0.68	–	–	1.10	5.84	–
IM2M	–1494.24	3,004.48	3.29E−58	13,645.78	0.27	0.16	12.53	1.42E−04	0.02	0.11	–	0.43	0.62
AM2M	–1457.91	2,933.82	7.28E−43	5,320.46	1.07	0.28	18.63	17.02	0.01	0.20	2.00	0.08	0.47
SC2M	–1360.88	2,739.77	1.00	12,092.45	0.18	0.15	29.91	1.43E−03	0.14	0.10	0.06	0.25	0.55

Note

Theta: mutation parameter for the ancestral population defined as *θ* = 4*N*_ref_ *µL*, where *µ* is the mutation rate per nucleotide site per generation and *L* is the total length of the analysed polymorphic sequences; *N*_Ag_: effective population size for *A. gigas*; *N*_Ps_: effective population size for *P. soyoae*; *M*_Ag‐Ps_: migration from *P. soyoae* into *A. gigas* in units of 2*N*_ref_·*m*_Ag‐Ps_ generations, where *m*_Ag‐Ps_ is the fraction of migrants from *P. soyoae* to *A. gigas* each generation; *M*_Ps‐Ag_: migration from *A. gigas* into *P. soyoae* in units of 2*N*_ref_·*m*_Ps‐Ag_ generations, where *m*_Ps‐Ag_ is the fraction of migrants from *A. gigas* to *P. soyoae* each generation; Me_Ag‐Ps_: effective migration rate of genes under selection from *P. soyoae* to *A. gigas*; Me_Ps‐Ag_: effective migration rate of genes under selection from *A. gigas* to *P. soyoae*; *T*_am_: scaled time between species split and end of ancient migration in units of 2*N*_ref_ generations; *T*_sc_: scaled time between secondary contact and present in units of 2*N*_ref_ generations; *T*_s_: scaled time between species split and present (IM), species split and secondary contact (SC) or end of ancient migration and present (AM) in units of 2*N*_ref_ generations; *P*: proportion of the genome evolving neutrally.

Abbreviations: AIC, Akaike information criterion; AICw, AIC weights; Ln, model likelihood.

## 4 DISCUSSION

Obligately vertical transmission results in co‐inheritance of symbionts with the mitochondrial genome of the host. Under this scenario, genetic coupling and ultimately cospeciation of host and symbiont lineages are expected, unless symbionts are occasionally transferred between host species (reviewed in Vrijenhoek, 2010). In vesicomyid clams, several instances of symbiont leakage have been reported by previous studies (Decker et al., 2013; Ozawa et al., 2017; Stewart & Cavanaugh, 2009; Stewart et al., 2008; Stewart, Young, & Cavanaugh, 2009), but the underlying mechanisms remain poorly understood. Stewart et al. (2008) suggested three different circumstances under which lateral symbiont transfer could occur: (a) host hybridization, (b) environmental acquisition from a free‐living symbiont population or (c) host‐to‐host transfer, for example through direct contact between symbiont‐bearing eggs or uptake of symbiont cells that have been released from a dying clam individual. Although the host‐to‐host transfer hypothesis has been favoured by multiple authors (Decker et al., 2013; Ikuta et al., 2016; Ozawa et al., 2017; Stewart et al., 2008), the possibility of interspecific hybridization has never been investigated.

Hybridization between different species is an important evolutionary process that can provide fundamental insights into the molecular mechanisms of reproductive isolation and adaptation. The outcomes of interspecific gene flow can be seen as a continuum of two extremes: erosion of species barriers through merging of gene pools (Allendorf, Leary, Spruell, & Wenburg, 2001) or evolution of new species through novel adaptive trait combinations in hybrids (Gompert, Fordyce, Forister, Shapiro, & Nice, 2006; Marques, Meier, & Seehausen, 2019; Seehausen, 2004, 2013). Recent studies have emphasized the importance of symbiotic microbes in animal speciation, with particular focus on their roles in hybrid incompatibility and reinforcement of existing species boundaries (Brucker & Bordenstein, 2012). While this emerging concept highlights the interactions between hybridization and symbiosis in evolution, synergistic effects of these two mechanisms in adaptive trait introgression and hybrid speciation are unknown.

In this study, we examined the symbiont composition and host hybridization hypothesis in the two clam species, *A. gigas* and *P. soyoae*, from cold seep sites in the Pacific Ocean. Our barcoding analyses indicated that several individuals with the *A. gigas* mitotype contained the *P. soyoae*‐specific symbiont phylotype at localities where both host species either co‐occurred (Ben's Seep and Extrovert Cliff) or where *A. gigas* was the only taxon found (Pedro's Whalefall). Demographic inference provided evidence that asymmetric gene flow between *P. soyoae* and *A. gigas* did occur in the evolutionary history of the two species. Both ima2 and ∂a∂i analyses favoured a model of mostly unidirectional migration from *P. soyoae* to *A. gigas* over models of strict isolation. Based on genomic information, ∂a∂i identified a secondary contact scenario with differential gene introgression as best fit to the observed joint allele frequency spectrum. The SC2M model is the most likely demographic scenario, among those that we examined, underlying the evolutionary divergence of these clam species. Interestingly, however, we did not find any evidence for admixed individuals in the investigated clam populations, as might be expected under a recent secondary contact scenario. This observation could be due to insufficient sampling. It is also possible that there have been enough generations that the genetic disequilibria from a recent hybridization event have nearly reached equilibrium.

Although open questions about the demographic history of *A. gigas* and *P. soyoae* remain, our data suggest that interspecific hybridization could be a mechanism for horizontal symbiont transmission and might explain occurrences of the *P. soyoae* symbiont phylotype in *A. gigas*‐like clam hosts. Surprisingly, we did not observe the alternative combination, that is *P. soyoae* hosts with the *A. gigas* symbiont. A simple technical explanation for this phenomenon could be sampling gaps, which are notorious problems in deep‐sea research. A possible biological explanation for our findings that is in line with the outputs of the SC2M model is asymmetric genetic incompatibilities between host species and symbionts that result in strong selection against the *P. soyoae* host × *A. gigas* symbiont combination. Vertical transmission usually leads to co‐adaptation of host–symbiont gene complexes, which are often disrupted through interspecific hybridization (Bordenstein, O'Hara, & Werren, 2001; Bordenstein & Werren, 2007; Brucker, & Bordenstein, 2012, 2013; Jaenike, Dyer, Cornish, & Minhas, 2006; Vala, Breeuwer, & Sabelis, 2000). Under this scenario, the *P. soyoae* symbiont would have an unknown fitness advantage that favours switching to a new host.

Incongruent compositions of host mitochondrial and symbiont genomes due to hybridization must involve some form of paternal cotransmission. One possibility is occasional inheritance of mitochondria and symbionts through the paternal germline (paternal leakage). Paternal leakage is a common phenomenon that occurs in a variety of different taxa at low frequency (reviewed in Breton & Stewart, 2015), which would agree with our observation that introgressed symbionts are rare. The second possibility is doubly uniparental inheritance (DUI) of mitochondria, which would involve a regulated system of paternal cotransmission as suggested previously for *Vesicomya* sp. mt‐II (Stewart et al., 2008). Although DUI has not been described in vesicomyid clams, it is known to occur in some Veneroida (Gusman, Lecomte, Stewart, Passamonti, & Breton, 2016; Zouros, 2013). To determine whether DUI is present in vesicomyids, it would be necessary to sequence mitochondrial genomes from sexed individuals and identify if genetic differences exist between female and male mitochondria.

Despite supporting hybridization as a potential mechanism of lateral symbiont transfer, our data do not reject the alternative hypotheses that were stated in the literature, although those seem less likely in this case for several reasons. First, the symbionts of *A. gigas* and *P. soyoae* belong to vesicomyid symbiont Clade I which is characterized by highly reduced genomes without essential genes for an extracellular lifestyle (Kuwahara et al., 2011), so that it is improbable that a free‐living population of these symbionts exists in the environment. Second, if physical proximity promoted lateral symbiont acquisition as argued by Decker et al. (2013), we would expect to find more individuals with non‐native symbionts in sympatric populations of *A. gigas* and *P. soyoae*. Furthermore, non‐native symbiont phylotypes were only observed in *A. gigas*, but not in *P. soyoae*. If host‐to‐host transfer was the underlying mechanism leading to symbiont switching, foreign phylotypes should be observed in both species, unless natural selection acted against the *P. soyoae* host × *A. gigas* symbiont combination (as mentioned above).

Our results raise several interesting hypotheses that can be addressed in future studies. In this study, we only investigated the symbiont *16S* rRNA gene. To disentangle host‐to‐host transfer from retention of an introgressed strain, it will be necessary to sequence and compare whole genomes of symbionts from clam individuals that contain the native and foreign *P. soyoae* phylotypes. A symbiont that was transferred historically via host hybridization can be expected to be highly divergent from the native phylotype, while a symbiont that was transmitted via contemporary host‐to‐host transfer should be relatively similar. Since we sequenced the *16S* rRNA gene directly, we were not able to uncover potential symbiont mixtures in individual host animals, given that this sequencing approach is biased towards the most abundant phylotype (Zimmermann et al., 2014). Furthermore, symbiont types could be variable across the host gill, as seen in other taxa from chemosynthetic environments (Duperron et al., 2005; Zimmermann et al., 2014). Studies that involved cloning and pyrosequencing techniques showed that in some cases divergent symbiont lineages can co‐occur in a single clam host (Decker et al., 2013; Stewart & Cavanaugh, 2009). To examine whether and how different symbiont lineages coexist in *A. gigas* and *P. soyoae*, whole‐genome analyses based on high‐throughput sequencing techniques will be useful. Comparative genomic and population genomic approaches will help to illuminate the genomic consequences of occasional lateral symbiont acquisition in deep‐sea vesicomyid clams and lead to a better understanding of how host–symbiont interactions shape the evolution of both partners.

## AUTHOR CONTRIBUTIONS

C.B. and S.B.J. designed the study. C.B. performed the RADseq, bioinformatic and statistical analyses and wrote the manuscript. S.B.J. found the mismatched symbiont strains by Sanger sequencing, produced the graphics and conducted the statistical analyses for the Sanger sequenced genes. C.R.Y. and R.C.V. advised on the study concept and statistical analyses. All authors contributed to writing the manuscript and agreed to this version of the manuscript.

## Data Availability

Sanger sequenced reads for *16S*, *mtCOI*, *H3* and *ANT* have been uploaded to GenBank (https://www.ncbi.nlm.nih.gov/genbank/) under Accession nos MK060220–MK060689. Raw ezRAD data have been deposited in the Sequence Read Archive (https://www.ncbi.nlm.nih.gov/sra) under BioProject number PRJNA497587. Bioinformatic code for the ezRAD analyses is provided in the Appendix S1–S6.
