# Supplementary material for: Host hybridization as a potential mechanism of lateral symbiont transfer in deep‐sea vesicomyid clams
Source: Mol Ecol. 2019 Sep 23;28(21):4697–708. doi: 10.1111/mec.15224 (PMC7004080; doi:10.1111/mec.15224)

MOLECULAR ECOLOGY

Supplemental Information for:

Host hybridization as a potential mechanism of lateral symbiont transfer in deep-sea vesicomyid clams

Corinna Breusing, Shannon B. Johnson, Robert C. Vrijenhoek, C. Robert Young

Appendix S1: ezRAD library preparation protocol

A. Fragment DNA

1. Measure the concentration of the DNA sample using a Qubit assay.

2. Add 100 to 500 ng of genomic DNA to a new PCR tube. Adjust the volume to 30 µl with 10 mM Tris-HCl pH 8.5 (or Qiagen Buffer EB).

3. Prepare FatI reaction mix with 0.1X overage by combining the following reagents:

   - 10 µl water
   - 5 µl 10X NEB Buffer 2.1
   - 5 µl FatI Restriction Enzyme

4. Gently vortex the Master Mix and spin briefly in a microfuge.

5. Add 20 µl of FatI Reaction Mix to each genomic DNA sample for a final volume of 50 µl.

6. Place the tubes on an Eppendorf MixMate and mix for 30 seconds at 2000 rpm. Briefly spin contents down by centrifugation.

7. Transfer tubes to a thermal cycler with heated lid set at 100°C and run the profile "ER2":

   - 55°C Pre-Heat Hold
   - 55°C for 30 minutes
   - Hold at 4°C

8. Immediately proceed to “Purify FatI-digested DNA”.

B. Purify FatI-digested DNA

FatI digested DNA is purified using a Zymo Research Genomic DNA Clean and Concentrator Kit.

1. Transfer the FatI-digested DNA to a 1.5 ml Eppendorf tube.
2. Add 250 µl (5 volumes) of DNA Binding Buffer to each sample.
3. Briefly vortex followed by centrifugation in a microfuge.
4. Load each sample onto a Zymo Genomic DNA Clean and Concentrator spin column.
5. Centrifuge the column at 13,000 rpm for 30 seconds.
6. Remove and discard the flow-through.
7. Add 200 µl of DNA Wash Buffer to each column.
8. Centrifuge the column at 13,000 rpm for 30 seconds.
9. Remove and discard the flow-through.
10. Add an additional 200 µl of DNA Wash Buffer to each column.
11. Centrifuge the column at 13,000 rpm for 2 minutes.
12. Remove and discard the flow-through.
13. Transfer the column to a new 1.5 ml Eppendorf tube.
14. Add 37 µl of Illumina Resuspension Buffer (10 mM Tris-HCl pH 8.5) to each sample.
15. Incubate at room temperature for 1 minute.
16. Centrifuge the column at 13,000 rpm for 30 seconds.
17. Immediately proceed to “End Repair”.

C. End Repair

Library Preparation is performed using the TruSeq Nano DNA Library Prep Kit (FC-121-4003).
Bring the following items to room temperature:
Sample Purification Beads (vigorously vortex before each use)

Resuspension Buffer (RB; can be stored at 2°C to 8°C after initial thaw)
End Repair Mix 2 (thaw at room temperature and then place in ice block)
Following the initial thaw of End Repair Mix 2, dispense the mix into aliquots of 200 µl to minimize the number of freeze thaw cycles that the reagent experiences.

1. Gently rock End Repair Mix 2 back and forth several times and then briefly spin in a microfuge.

2. Add 24 µl of End Repair Mix 2 to each purified, FatI-digested DNA sample (36 µl) for a total volume of 60 µl.

3. Place the tubes on an Eppendorf MixMate and mix for 30 seconds at 2000 rpm. Briefly spin contents down by centrifugation.

4. Transfer tubes to a thermal cycler with heated lid set at 100°C and run the profile "ER2":

   - 30°C Pre-Heat Hold
   - 30°C for 30 minutes
   - Hold at 4°C

5. Immediately proceed to “Size Selection of End Repaired DNA”.

D. Size Selection of End Repaired DNA (average 400 bp)

1. Prepare a mixture of 67% Sample Purification Beads by combining the following volumes of reagents for each reaction. Vortex the Sample Purification Beads well before and after preparing the diluted bead mixture. Prepare 5% overage when processing multiple samples:

   - 67 µl of Sample Purification Beads
   - 33 µl of water

2. Add 96 µl of the 67% Sample Purification Bead Mixture to a 1.5 ml Eppendorf tube.

3. Transfer the 60 µl End Repair reaction to the tube containing diluted Sample Purification Beads.

4. Vortex well and incubate at room temperature for 5 minutes.

5. Following brief centrifugation, place tubes on a magnetic stand for 2 minutes.

6. Carefully transfer 150 µl of supernatant to a new 1.5 ml Eppendorf tube containing 15 µl of undiluted Sample Purification Beads. Avoid the transfer of beads from the magnetic pellet. Following transfer, you can discard the tube containing the magnetic pellet.

7. Vortex the mixture well and incubate at room temperature for 5 minutes.

8. Following brief centrifugation, place tubes on a magnetic stand for 2 minutes.

9. Remove approximately 160 µl of supernatant and discard.

10. Wash the magnetic pellet 2X with 180 µl of 80% EtOH while leaving the tubes on the magnetic stand. Following the second wash, use a p10 pipettor to remove any residual EtOH in the tube.

11. Allow the magnetic pellet on side of tube to dry for approximately 5 minutes until small cracks appear in the pellet.

12. Add 12 µl of RB to the dried pellet and vortex to resuspend the pellet.

13. Incubate at room temperature for 2 minutes.

14. Following brief centrifugation, transfer the tubes to a magnetic stand for 2 minutes.

15. Transfer 10.5 µl of supernatant to a new PCR tube.

16. If you do not plan to immediately perform "Adenylate 3' Ends", you can safely store the end-repaired DNA at –20°C for up to one week.

E. Adenylate 3' Ends

Bring the following items to room temperature:
Sample Purification Beads (vigorously vortex before each use)
Resuspension Buffer (can be stored at 2°C to 8°C after initial thaw)
A-Tailing Mix should be thawed at room temperature and then placed in an ice block
Following the initial thaw of the A-Tailing Mix, dispense the mix into aliquots of 150 µl to minimize the number of freeze thaw cycles that the reagent experiences

1. Combine 7.5 µl of A-Tailing Mix with the purified, end-repaired DNA for a total volume of 18 µl.

2. Place the sample tubes on an Eppendorf MixMate and mix for 60 seconds at 2000 rpm. Briefly spin contents down by centrifugation.

3. Transfer the tubes to a thermal cycler and run the profile "A-TAIL70" with heated lid set at 75°C to perform adenylation of 3' ends:

   - 37°C Pre-Heat Hold
   - 37°C for 30 minutes
   - 70°C for 5 minutes
   - Hold at 4°C

4. When the thermal cycler reaches 4°C, remove the tubes from the thermal cycler and proceed immediately to "Adapter Ligation".

F. Adapter Ligation

Remove RNA Adapter Indexes from the freezer and thaw at room temperature. Briefly centrifuge. Immediately before use, remove Ligation Mix 2 from the freezer and transfer to an ice block to keep the mix cold.

1. Prepare Ligation Master Mix by combining the following volumes of reagents for each reaction (prepare 20% overage when processing multiple samples):

   - 1.5 µl Resuspension Buffer
   - 1.5 µl Ligation Mix 2

2. After combining the reagents, vortex the Master Mix and spin contents down by brief centrifugation.

3. Add 3.0 µl of the Ligation Master Mix to each tube.

4. Add 1.5 µl of Indexed Adapter to each tube for a final volume of 22.5 µl (samples in the same multiplex group must contain unique indices).

5. Place the tubes on an Eppendorf MixMate and mix for 60 seconds at 2000 rpm. Briefly spin contents down by centrifugation.

6. Transfer the tubes to a thermal cycler and run the profile "Ligate" with heated lid set at 100°C to ligate adapters to 3'-adenylated cDNA:

   - 30°C Pre-heat Hold
   - 30°C for 10 minutes
   - 4°C Hold

7. When the thermal cycler reaches 4°C, remove the tubes from the thermal cycler and immediately proceed to “Purification of Adapter-Ligated DNA”.

G. Purification of Adapter-Ligated DNA

1. Remove reaction tubes from the thermal cycler and place at room temperature.

2. Prepare a mixture of Stop Ligation Buffer and AMPure XP beads by combining the following volumes for each reaction (make 10% overage per reaction).

    - 3 µl Stop Ligation Buffer
    - 25 µl of Sample Purification Beads

3. For each sample, add 28 µl of Stop Ligation Buffer/Sample Purification Beads mixture to a 1.5 ml Eppendorf tube.

4. Transfer the adapter-ligated DNA sample to the tube containing the Sample Purification Beads mixture and mix the contents by vortex.

5. Incubate at room temperature for 5 minutes.

6. Following brief centrifugation, place tubes on a magnetic stand for 2 minutes.

7. Remove approximately 45 µl of supernatant and discard.

8. Wash the magnetic pellet 2X with 180 µl of 80% EtOH while leaving the tubes on the magnetic stand. Following the second wash, use a p10 pipettor to remove any residual EtOH in the tube.

9. Allow the magnetic pellet to dry for approximately 5 minutes until small cracks appear in the pellet.

10. Add 32 µl of RB to the dried pellet and vortex to resuspend the pellet.

11. Incubate at room temperature for 2 minutes.

12. Following brief centrifugation, transfer the tubes to a magnetic stand for 2 minutes.

13. Transfer 30 µl of supernatant to a new 1.5 ml Eppendorf tube containing 30 µl of undiluted Sample Purification Beads (it is OK if residual beads are transferred with the sample).

14. Vortex the tubes to mix and incubate at room temperature for 5 minutes.

15. Following brief centrifugation, transfer the tubes to a magnetic stand for 2 minutes.

16. Remove approximately 55 µl of supernatant and discard.

17. Wash the magnetic pellet 2X with 180 µl of 80% EtOH while leaving the tubes on the magnetic stand. Following the second wash, use a p10 pipettor to remove any residual EtOH in the tube.

18. Allow the magnetic pellet to dry for approximately 5 minutes until small cracks appear in the pellet.

19. Add 17 µl of RB to the dried pellet and vortex to resuspend the pellet.

20. Incubate at room temperature for 2 minutes.

21. Following brief centrifugation, transfer the tubes to a magnetic stand for 2 minutes.

22. Transfer 15 µl of supernatant to a new PCR tube in preparation to enrich DNA fragments by PCR.

23. If you do not plan to immediately perform "Enrich DNA Fragments by PCR", you can safely store the adapter-ligated DNA at –20°C for up to one week.

H. Enrich DNA Fragments by PCR

Bring the following items to room temperature:
Sample Purification Beads (vigorously vortex before each use)
Resuspension Buffer (can be stored at 2°C to 8°C after initial thaw)
PCR Primer Cocktail (thaw at room temperature and then spin contents down by brief centrifugation)
Enhanced PCR Mix (thaw at room temperature and then transfer the tube to ice block)
Following the initial thaw of Enhanced PCR Mix, dispense the mix into aliquots of 200 µl to minimize the number of freeze thaw cycles that the reagent experiences

1. Prepare PCR Master Mix by combining the following volumes of reagents for each reaction (prepare 5% overage when processing multiple samples):

    - 12 µl of Enhanced PCR Mix
    - 3 µl of PCR Primer Cocktail

2. Add 15 µl of the PCR Master Mix prepared in step 1 to each sample of adapter-ligated DNA for a final volume of 30 µl. (Alternatively, purified adapter-ligated cDNA can be added directly to PCR tubes that already contain the PCR Reaction Mix).

3. Place the tube on an Eppendorf MixMate and mix for 30 seconds at 2000 rpm. Briefly spin contents down by centrifugation.

4. Transfer the reaction tube to a thermal cycler with a heated lid set at 100°C and run the profile "DNA-6C":

    - 98°C for 30 seconds
    - 6 cycles of:
      --98°C for 20 seconds
      --60°C for 15 seconds
      --72°C for 30 seconds
    - 72°C for 5 minutes
    - Hold at 4°C

5. Immediately proceed to “Purification of PCR-Amplified QC Library”.

I. Purification of PCR-Amplified QC Library

1. For each sample, add 30 µl of Sample Purification Beads to a 1.5 ml Eppendorf tube.

2. Transfer the PCR-amplified sample to the tube containing Sample Purification Beads and mix the contents by vortexing.

3. Incubate at room temperature for 5 minutes.

4. Following brief centrifugation, place tubes on a magnetic stand for 2 minutes.

5. Remove approximately 55 µl of supernatant and discard.

6. Wash the magnetic pellet 2X with 180 µl of 80% EtOH while leaving the tubes on the magnetic stand. Following the second wash, use a p10 pipettor to remove any residual EtOH in the tube.

7. Allow the magnetic pellet to dry for approximately 5 minutes until small cracks appear in the pellet.

8. Add 22 µl of RB to the dried pellet and vortex to resuspend the pellet.

9. Incubate at room temperature for 2 minutes.

10. Following brief centrifugation, transfer the tubes to a magnetic stand for 2 minutes.

11. Transfer 20 µl of supernatant to a new 1.5 ml Eppendorf tube.

MOLECULAR ECOLOGY

12. Measure the concentration of the library using a Qubit High Sensitivity dsDNA assay.

Appendix S2: Script for filtering of sequence contaminants

```
#!/bin/bash

FILE=("D97_1 D97_2 D97_3 D97_4 D97_8 T349_1 T349_2 T349_3 T349_4 T349_5 D474_1
D474_2 D474_3 D474_4")

bwa index contaminants.fasta

for file in ${FILE}
do
bwa mem -t 10 contaminants.fasta ${file}.F.fq.gz ${file}.R.fq.gz > ${file}.sam
samtools view -Sb -h -@ 10 -o ${file}.bam ${file}.sam
samtools sort -@ 10 -o ${file}.sorted.bam ${file}.bam
samtools view -f12 ${file}.sorted.bam > ${file}.unmapped.sam
cut -f1 ${file}.unmapped.sam | sort | uniq > ${file}.unmapped_ids.lst
/usr/local/bin/seqtk/seqtk subseq ${file}.F.fq.gz ${file}.unmapped_ids.lst > ${file}_1.F.fq
gzip ${file}_1.F.fq
/usr/local/bin/seqtk/seqtk subseq ${file}.R.fq.gz ${file}.unmapped_ids.lst > ${file}_1.R.fq
gzip ${file}_1.R.fq
done
```

Appendix S3: Basic quality metrics and information for the ezRAD sequencing reads

Name	Species	# raw reads	# filtered reads	# reads mapped to RAD assembly	% raw reads with Q ≧ 30	Length
D474_1	Hybrid *A. gigas*	75,799,934	64,388,342	19,150,843	90.42	2x150
D474_2	Hybrid *A. gigas*	76,598,474	68,052,228	22,653,960	91.13	2x150
D474_3	Hybrid *A. gigas*	86,358,368	75,326,810	23,392,340	89.57	2x150
D474_4	Hybrid *A. gigas*	78,696,658	68,064,710	16,134,792	87.29	2x150
T349_1	*A. gigas*	35,385,682	32,384,626	5,410,644	93.42	2x125
T349_2	*A. gigas*	14,591,566	13,519,964	2,274,801	92.15	2x125
T349_3	*A. gigas*	18,437,384	17,164,290	2,662,467	91.39	2x125
T349_4	*A. gigas*	21,492,094	19,793,470	3,111,806	92.25	2x125
T349_5	*A. gigas*	14,141,136	13,272,478	2,263,803	92.20	2x125
D97_1	*P. soyoae*	75,569,720	71,017,692	26,363,930	89.70	2x150
D97_2	*P. soyoae*	70,139,300	64,480,122	22,269,106	90.02	2x150
D97_3	*P. soyoae*	73,521,458	69,635,678	25,690,364	89.49	2x150
D97_4	*P. soyoae*	66,677,776	63,066,260	23,775,394	89.95	2x150
D97_8	*P. soyoae*	77,189,994	73,786,210	28,112,667	90.46	2x150

MOLECULAR ECOLOGY

Appendix S4: Script for genotype likelihood analyses in ANGSD

```
#!/bin/bash
#SBATCH -J ANGSD
#SBATCH -t 48:00:00
#SBATCH -n 90
#SBATCH --mem=50g
#SBATCH -o ANGSD.out
#SBATCH -e ANGSD.err

module load angsd

angsd -gl 1 -bam gigas.list -dosaf 1 -baq 1 -C 50 -minMapQ 30 -minQ 20 -P 90 -minInd 9   /
-setMinDepth 20 -setMaxDepth 250 -doCounts 1 -anc reference.fasta -ref reference.fasta   /
-uniqueOnly 1 -remove_bads 1 -only_proper_pairs 1 -out gigas

angsd -gl 1 -bam soyoae.list -dosaf 1 -baq 1 -C 50 -minMapQ 30 -minQ 20 -P 90 -minInd 5   /
-setMinDepth 20 -setMaxDepth 250 -doCounts 1 -anc reference.fasta -ref reference.fasta   /
-uniqueOnly 1 -remove_bads 1 -only_proper_pairs 1 -out soyoae

/gpfs/runtime/opt/angsd/0.920/angsd/misc/realSFS gigas.saf.idx soyoae.saf.idx -maxIter 100   /
-P 90 > soyoae_gigas.sfs

angsd -P 90 -bam bamlist.list -ref reference.fasta -out clams -uniqueOnly 1 -remove_bads 1   /
-only_proper_pairs 1 -C 50 -baq 1 -minMapQ 30 -minQ 20 -minInd 14 -setMinDepth 20   /
-setMaxDepth 250 -doCounts 1 -GL 1 -doMajorMinor 1 -doMaf 1 -doGeno 32 -doPost 1   /
-doGlf 2

/gpfs/data/rbeinart/Software/ngsTools/ngsPopGen/ngsCovar -probfile clams.geno   /
-outfile clams.covar -nind 14 -nsites 349288 -call 0 -norm 0

Rscript -e 'write.table(cbind(seq(1, 14), rep(1, 14), c(rep("Hybrid A. gigas", 4),   /
rep("P. soyoae", 5), rep("A. gigas", 5))), row.names = F, sep = "\t", col.names = c("FID",   /
"IID", "CLUSTER"), file = "clams.clst", quote = F)'

Rscript plotPCA.R -i clams.covar -c 1-2 -a clams.clst -o clams.pca.pdf

/gpfs/runtime/opt/angsd/0.920/angsd/misc/NGSadmix -likes clams.beagle.gz -K 2   /
-outfiles clams_admix2 -P 90 -minMaf 0 -maxiter 100000

/gpfs/runtime/opt/angsd/0.920/angsd/misc/NGSadmix -likes clams.beagle.gz -K 3   /
-outfiles clams_admix3 -P 90 -minMaf 0 -maxiter 100000
```

MOLECULAR ECOLOGY

Appendix S5: Pairwise F_{ST} values for mitochondrial (below diagonal) and nuclear (above diagonal) Sanger sequence data. Bold values indicate significant differences after Benjamini-Yekutieli false discovery rate correction. Note that in the case of GoC sample sizes were small and pairwise differences were therefore insignificant. GoC = GoC Site#7, BS = Ben's Seep, PW = Pedro's Whalefall, GR = Gorda Ridge, EC = Extrovert Cliff, SDF = San Diego Fault, CB = Clam Bed.

		A. gigas					*P. soyoae*				
		**GoC**	**BS**	**PW**	**GR**	**EC**	**GoC**	**BS**	**SDF**	**CB**	**EC**
A. gigas	**GoC**	*	0.098	0.103	0.059	0.159	0.266	0.321	0.215	0.169	0.227
	**BS**	0.000	*	–0.004	–0.001	**0.106**	**0.276**	**0.305**	**0.266**	**0.213**	**0.231**
	**PW**	–0.070	0.033	*	–0.010	**0.111**	**0.292**	**0.317**	**0.283**	**0.233**	**0.252**
	**GR**	–0.026	0.097	–0.013	*	0.055	**0.225**	**0.262**	**0.208**	**0.157**	**0.192**
	**EC**	0.008	0.133	–0.004	–0.083	*	**0.168**	**0.176**	**0.142**	0.077	**0.110**
P. soyoae	**GoC**	1.000	**1.000**	**0.704**	**0.748**	**0.647**	*	–0.076	–0.102	–0.044	–0.003
	**BS**	**1.000**	**1.000**	**0.808**	**0.856**	**0.790**	0.000	*	–0.068	–0.019	0.006
	**SDF**	1.000	**1.000**	**0.704**	**0.748**	**0.647**	0.000	0.000	*	–0.084	–0.054
	**CB**	**0.797**	**0.868**	**0.626**	**0.653**	**0.561**	–0.081	0.167	–0.081	*	–0.063
	**EC**	**0.908**	**0.926**	**0.762**	**0.798**	**0.737**	–0.136	–0.011	–0.136	–0.036	*

MOLECULAR ECOLOGY

Appendix S6: Admixture analyses in NGSadmix based on A) K=2 and B) K=3.

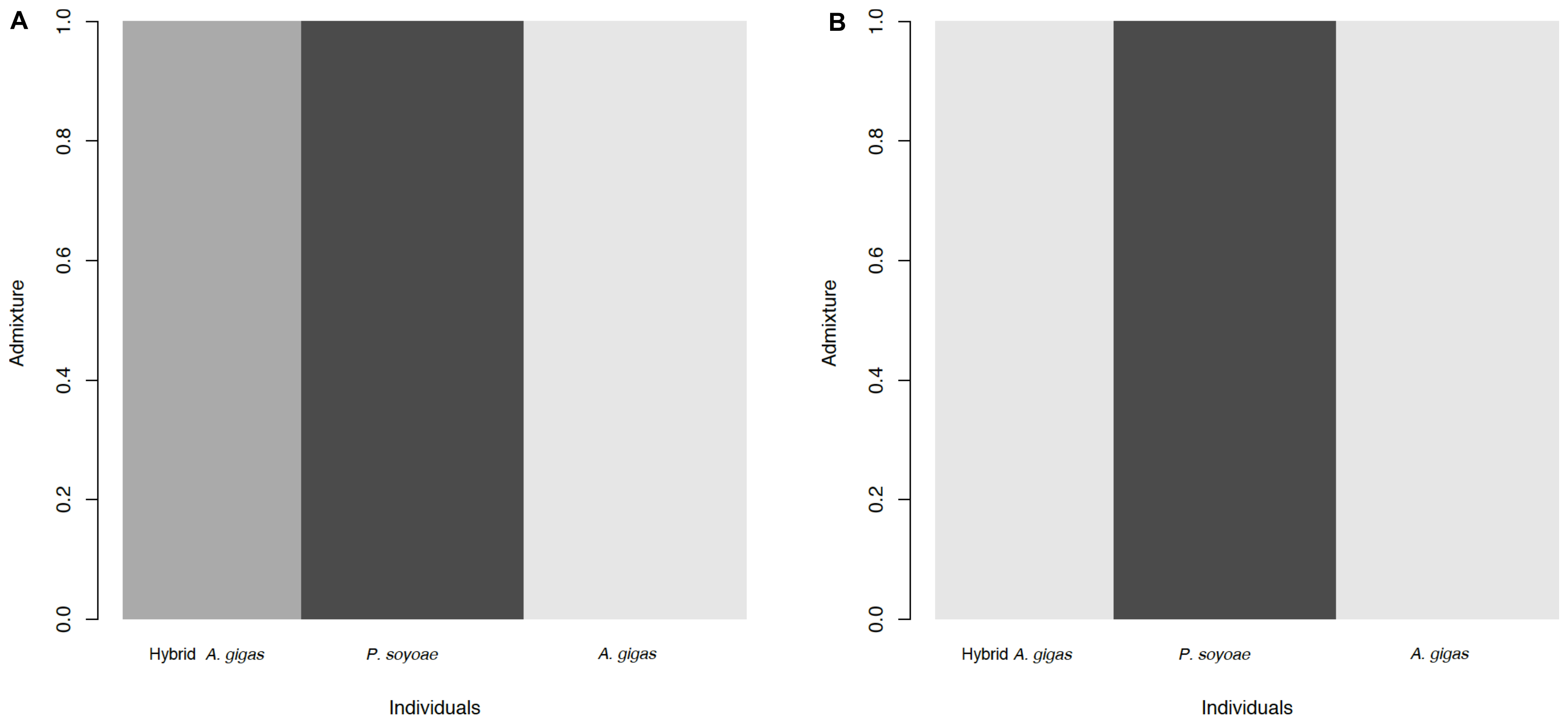

Supplement: Supplementary file 1 [file MEC-28-4697-s001.pdf]
